# INNOVATE: A prospective cohort study combining serum and urinary biomarkers with novel diffusion-weighted magnetic resonance imaging for the prediction and characterization of prostate cancer

**DOI:** 10.1186/s12885-016-2856-2

**Published:** 2016-10-21

**Authors:** Edward Johnston, Hayley Pye, Elisenda Bonet-Carne, Eleftheria Panagiotaki, Dominic Patel, Myria Galazi, Susan Heavey, Lina Carmona, Alexander Freeman, Giorgia Trevisan, Clare Allen, Alexander Kirkham, Keith Burling, Nicola Stevens, David Hawkes, Mark Emberton, Caroline Moore, Hashim U Ahmed, David Atkinson, Manuel Rodriguez-Justo, Tony Ng, Daniel Alexander, Hayley Whitaker, Shonit Punwani

**Affiliations:** 1 UCL Centre for Medical Imaging, 5th floor, Wolfson House, 4 Stephenson Way, London, NW1 2HE UK; 2 Research Department for Tissue & Energy, Division of Surgery & Interventional Science, Wing 2.4 Cruciform Building, Gower Street, London, WC1E 6BT UK; 3 Addenbrookes Hospital, Level 4, Pathology Block, Hills Road, Cambridge, CB20QQ UK; 4 Department of Research Pathology, UCL Cancer Institute, Rockefeller Building, 21 University Street, London, WC1E 6JJ UK; 5 Department of Computer Science, UCL, Gower Street, London, WC1E 6BT UK; 6 Molecular Oncology group, UCL Cancer Institute, Paul O’Gorman Building, 72 Huntley Street, London, WC1E 6DD UK; 7 Division of Surgery, 4th floor, 21 University Street, London, WC1E UK

## Abstract

**Background:**

Whilst multi-parametric magnetic resonance imaging (mp-MRI) has been a significant advance in the diagnosis of prostate cancer, scanning all patients with elevated prostate specific antigen (PSA) levels is considered too costly for widespread National Health Service (NHS) use, as the predictive value of PSA levels for significant disease is poor. Despite the fact that novel blood and urine tests are available which may predict aggressive disease better than PSA, they are not routinely employed due to a lack of clinical validity studies.

Furthermore approximately 40 % of mp-MRI studies are reported as indeterminate, which can lead to repeat examinations or unnecessary biopsy with associated patient anxiety, discomfort, risk and additional costs.

**Methods/Design:**

We aim to clinically validate a panel of minimally invasive promising blood and urine biomarkers, to better select patients that will benefit from a multiparametric prostate MRI. We will then test whether the performance of the mp-MRI can be improved by the addition of an advanced diffusion-weighted MRI technique, which uses a biophysical model to characterise tissue microstructure called VERDICT; Vascular and Extracellular Restricted Diffusion for Cytometry in Tumours.

INNOVATE is a prospective single centre cohort study in 365 patients. mp-MRI will act as the reference standard for the biomarker panel. A clinical outcome based reference standard based on biopsy, mp-MRI and follow-up will be used for VERDICT MRI.

**Discussion:**

We expect the combined effect of biomarkers and VERDICT MRI will improve care by better detecting aggressive prostate cancer early and make mp-MRI before biopsy economically viable for universal NHS adoption.

**Trial registration:**

INNOVATE is registered on ClinicalTrials.gov, with reference NCT02689271.

**Electronic supplementary material:**

The online version of this article (doi:10.1186/s12885-016-2856-2) contains supplementary material, which is available to authorized users.

## Background

The management of prostate cancer poses difficult challenges, which is largely because we lack the necessary tools to predict its presence, and discern between indolent disease with a small chance of clinical manifestation and aggressive tumours that are more likely to be lethal. Since prostate cancer is a complex disease, it is unlikely to be fully characterised with a single fluidic or diagnostic imaging marker.

### The standard and our institutional diagnostic pathways

After presenting with symptoms, or requesting screening for prostate cancer, patients typically undergo a digital rectal exam (DRE), combined with a prostate-specific antigen (PSA) blood test.

### PSA

PSA is a glycoprotein enzyme produced by normal prostate epithelium and is routinely used as a serum biomarker for prostate cancer, with raised levels typically provoking trans rectal ultrasound (TRUS) biopsy. However, in addition to prostate cancer, raised PSA levels are encountered in benign prostatic hyperplasia (BPH), prostatitis and normal prostate tissue, the PSA test has a fairly flat receiver operator characteristic curve, resulting in false positive and negative results meaning it is relatively poor at predicting or excluding significant prostate cancer [1, 2], which drives the need for more specific circulating biomarkers in its diagnosis. Circulating biomarkers in serum, plasma, urine, and prostatic fluid have all been explored, but thus far remain invalidated to a defined standard in a cohort collected under standardised conditions.

### PCA3

PCA3 (prostate cancer antigen 3) is the only other routinely available biomarker, it is currently only available in a private healthcare setting. The PCA3 test is carried on urine out after DRE and detects a prostate specific non-coding ribonucleic acid (RNA). The test has shown clinical utility in diagnosing prostate cancer and can discriminate tumour cells from benign [3–5]. When used alongside magnetic resonance imaging (MRI) it shows a correlation with tumour volume but PCA3 does not appear to correlate with other clinical parameters such as stage and grade [6]. When used alongside MRI the accuracy of the PCA3 test can be improved, PCA3 score has also been shown to correlate with suspicious MRI findings and therefore could be used to select patients that require an MRI, or because MRI outperforms the PCA3 it may have greater utility in stratifying patients for active surveillance or further biopsy [7–9].

### MRI

In the last 5 years, the prostate cancer community has undergone a pivotal change away from random transrectal ultrasound (TRUS) sampling of the prostate and towards image guided biopsy requiring multiparametric (mp)-MRI, including T2 weighted (T2W), diffusion weighted (DWI) and often dynamic contrast enhanced (DCE) imaging.

In January 2014 the National Institute of Clinical Excellence (NICE) issued revised guidelines on the management of prostate cancer, which included the use of mp-MRI in prostate cancer diagnostics [10]. In this document, MRI was only recommended in those with a negative TRUS and for staging where a change in tumour (T) or nodal (N) staging would alter management. The reason for this is likely to be due to the fact that mp-MRI remains a less than perfect test. For example, mp-MRI is relatively expensive, approximately 40 % of patients have equivocal findings and performance is modest for detection of small volume (<0.5 cc) tumour, lower grade aggressive disease (secondary Gleason pattern 4) and for lesions within the transition zone. In addition, the correlation of mp-MRI derived quantitative metrics with Gleason grade is only moderate; meaning it lacks biological specificity. This means further repeat multiparametric MRI studies or unnecessary biopsies are often necessitated, with associated patient discomfort, additional risks and costs.

## Our proposed new pathway

**Fig. 1:**
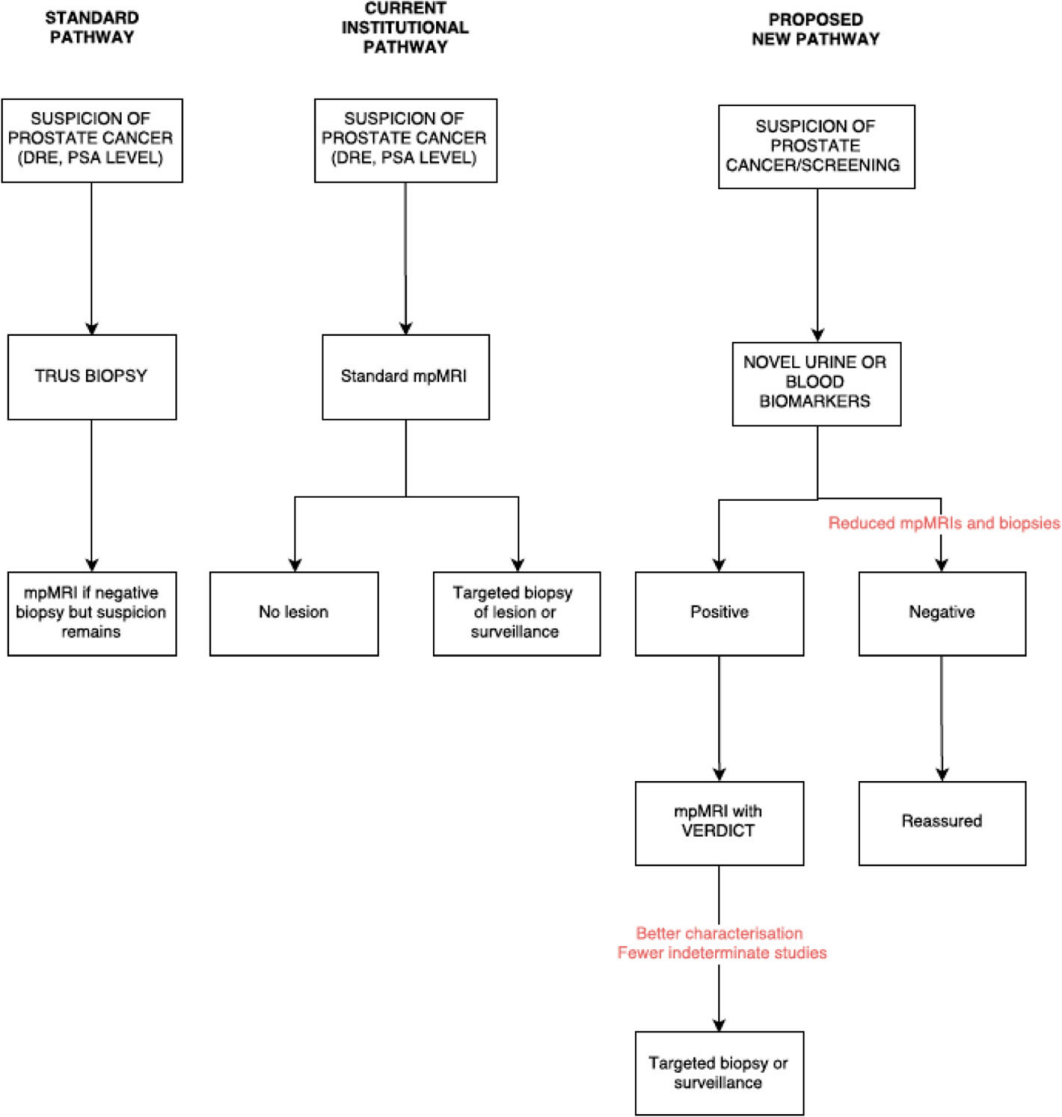
Standard, our institutional and proposed new pathways for prostate cancer diagnosis

To address these limitations, we propose an approach integrating promising fluidic markers together with advanced diffusion weighted MRI (VERDICT: Vascular, Extracellular and Restricted DIffusion for Cytometry in Tumours) within the diagnostic paradigm (Fig. 1).

### Novel serum and urine biomarkers

The fluidic biomarkers we propose to investigate in our study have been selected based on the number of studies, patient reports and the ability of a marker to discriminate tumour from benign or predict poor outcome (Additional file 1). All markers can be tested in minimally invasive samples e.g. whole blood, serum, plasma or urine. We envision that these markers would help select patients most likely to benefit from subsequent mp-MRI, thereby rationalising valuable NHS resources. Horizon scanning will continue throughout the study to include any new and promising markers.

### VERDICT MRI

Most diffusion-weighted MRI studies have used the technique in its simplest form by calculating the apparent diffusion coefficient (ADC) to identify clinically significant tumor foci more clearly [11, 12]. In general, ADC values are lower in prostate carcinoma compared with healthy tissue but ADC values in both tissue types vary widely and overlap substantially [12–14].

The recent VERDICT MRI technique [15] offers the potential for explicit characterisation of tissue histology non-invasively. A proof-of-concept study for assessment of human prostate cancer [16] provided the basis for a first-in-man study of clinical validity. In this study, we imaged 8 patients with histologically confirmed peripheral zone cancer and demonstrated significant elevation in tumour fractional intracellular and fractional vascular volume, and a reduction in fractional extracellular extravascular volume, in keeping with disease histology.

Since this work, the MRI protocol has been optimised, using a computational optimisation framework [17] to reduce the VERDICT scan time from 40 min to a more clinically acceptable time of 15 min.

This is the world’s first clinical trial to investigate the use of VERDICT MRI. We envision that application of VERDICT MRI will improve the specificity of mp-MRI, reduce the number of indeterminate examination results and provide evaluation of the specific histological feature changes associated with cancer.

## Methods and analysis

### Design

INNOVATE is a prospective cohort study with single centre recruitment. The primary objective is to assess whether supplementary VERDICT MRI improves the diagnostic accuracy of mp-MRI for detection of significant prostate cancer by a minimum of 10 %. The definitions of significant cancer have been provided previously [18].

Participants undergo standard mp-MRI [19] ± biopsy, together with studied index tests (fluidic markers and VERDICT MRI). A 50 patient pilot phase held over 1 year will provide histologically validated VERDICT MRI studies in order to familiarise radiologists and ascend the learning curve necessary for clinically interpreting VERDICT images. Initial evaluation of fluidic biomarker performance for prediction of a negative mp-MRI result will be conducted at the end of year 1 to derive thresholds for prospective application. An evaluation phase held over 2 years will prospectively test the added diagnostic accuracy of VERDICT to standard mp-MRI. During the evaluation phase, selected fluidic biomarker thresholds will be applied to collected samples to prospectively categorise patients into those expected to achieve negative and positive (with a lesion) mp-MRI scores.

### Patient population

Inclusion, exclusion and withdrawal criteria are provided in Table 1 below.

**Table 1:** Inclusion, exclusion and withdrawal criteria

Patient Inclusion Criteria
1. Men referred to our center for prostate mp-MRI following biopsy elsewhere
2. Biopsy naive men presenting to our institution with a clinical suspicion of prostate cancer
Patient Exclusion Criteria
1. Men unable to have a MRI scan, or in whom artifact would reduce quality of MRI
2. Men unable to given informed consent
3. Previous treatment (prostatectomy, radiotherapy, brachytherapy) of prostate cancer
4. On-going hormonal treatment for prostate cancer
5. Previous biopsy within 6 months of scheduled mp-MRI
Withdrawal criteria
1. Images inadequate for analysis due to artifact or image acquisition problems even after a repeat scan

### Informed consent

Informed consent is a prerequisite and will be carried out on the day of the trial interventions, following a minimum 24-h period of consideration to participate in the study.

### Trial Interventions

#### The index test – VERDICT MRI

All studies will be performed on a 3 T MRI scanner (Achieva, Philips, Amsterdam, NL). The total MRI protocol including routine mp-MRI will be limited to a maximum of 1-h scan time inclusive of 15 additional minutes allowed for VERDICT MRI. The mp-MRI protocol will be standardized, as recommended as per the UK consensus guidelines on prostate MRI [19], Table 2 below. 20 patients with tumours will undergo repeat studies, with one group having immediate repeatability (back to back scans) and another undergoing repeat studies within a week to gauge the short term repeatability of the parametric maps generated by VERDICT.

**Table 2:** MRI phasing details for standard multiparametric prostate MRI

Sequence	Coil	TR	TE	FA degrees	WFS(pix)	BW Hz/Px	Fov mm	Slice thickness mm	Gap	TSE factor	Phasing direction	FS	ACQ matrix	TFE shots	TFE shot interval (ms)	Total scan duration
T2 TSE coronal	Dual	6128	100	90	2.704	160.7	180	3	3	16	R > L	No	300 x 290			05:55.4
T2 TSE axial	Dual	5407	100	90	2.704	160.7	180	3	0	16	R > L	No	300 x 290			05:13.6
T2 sag REF	Dual	1579	100	90	1.999	217.3	240	5	5	20	A > P	No	120 x 89			00:18.9
T1W TSE	Dual	487	8	90	1.997	217.6	240	3	3	4	R > L	No	184 x 184			03:06.8
DWI 0 150 500 1000	Dual	2753	80	90	40.353	10.8	220	5	0		A > P	SPAIR	168 x 169			05:16.5
DWI b2000	Dual	2000	78	90	44.108	9.9	220	5	0		A > P	SPIR	168 x 169			03:40.0
DCE 2 dyn mod SENSE	Dual	5.8	2.8	90	1.766	246.1	180	3	0	38 (TFE)	R > L	SPAIR	140 x 177	49	280	00:28.9
DCE 20 dyn mod SENSE	Dual	5.8	2.8	90	1.766	246.1	180	3	0		R > L	SPAIR	140 x 162	45	280	04:14.1

This is supplemented by an optimised VERDICT MRI technique based on previously reported work [15], which uses a Pulse-gradient spin-echo sequence and a 32 channel cardiac coil with *b* values of 90-3000 s/mm^2^ in 3 orthogonal directions. For *b* < 500 the number of averages (NAV) = 6, for 500 < *b* < 1000 NAV = 12 and for *b* > 1000 NAV = 18 with voxel size = 1.3 × 1.3 × 5 mm, matrix size = 176 × 176. The data is normalized with a *b* = 0 image for every echo time (TE) to avoid T2 dependence. Scanning parameters for VERDICT MRI are provided in Table 3.

**Table 3:** VERDICT MRI diffusion gradient parameters

*b* value s/mm^2^	∆/δ ms	TE ms	|G| T/m
3000	19.7/38.8	80	0.0579
2000	13.2/32.3	67	0.0758
1500	24.7/43.8	90	0.0311
500	12.2/31.3	65	0.0415
90	4.7/23.8	50	0.0506

**Fig. 2:**
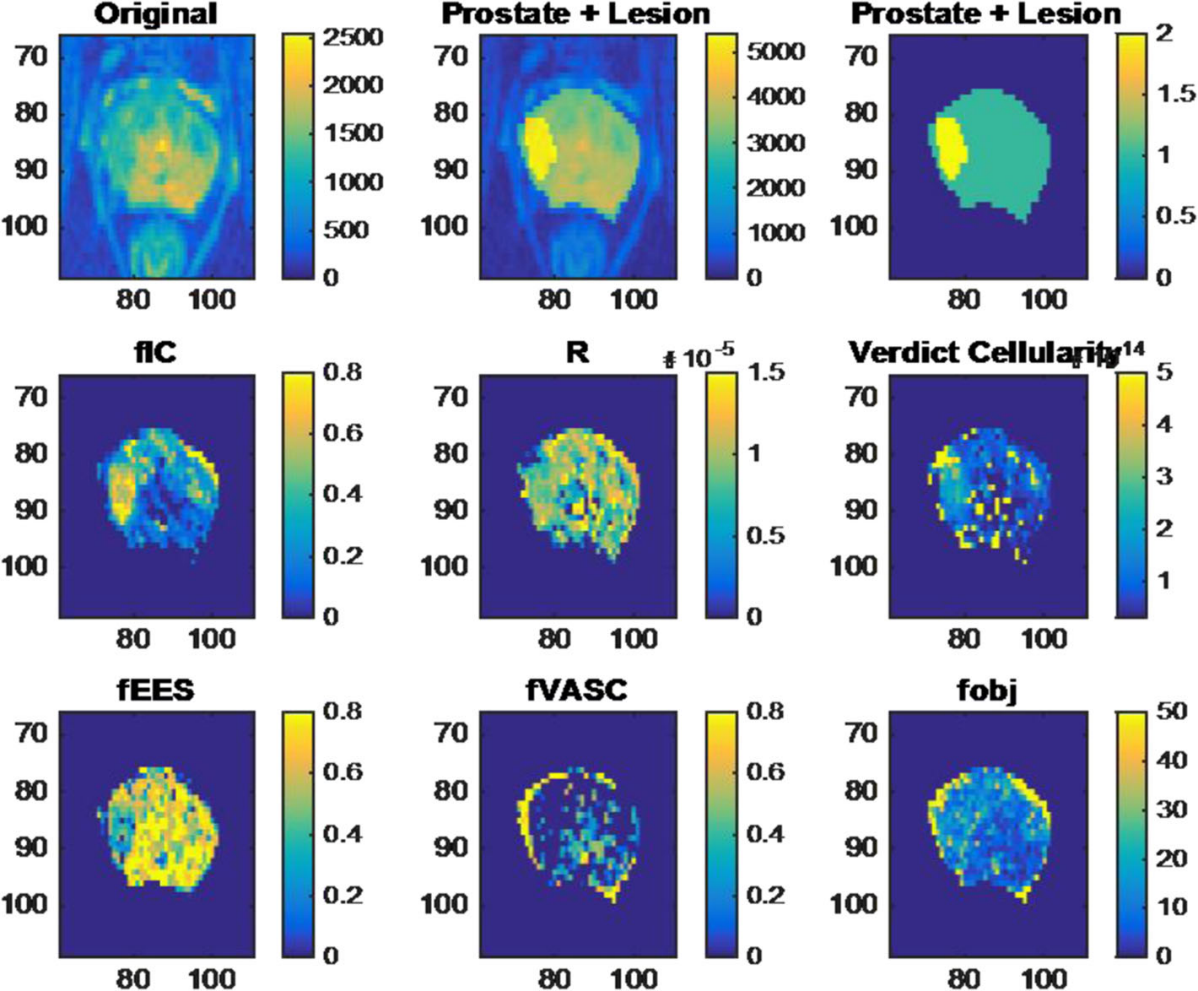
VERDICT parameter maps. Images have been colour scaled. L to R, top to bottom: Original image b = 0 diffusion-weighted image. Prostate + lesion showing original image with superimposed segmented lesion. Prostate segmentation + lesion segmentation. fIC = intracellular volume fraction. R = cell radius. Cellularity map = calculated parametric map which shows the measured number of cells per voxel. fEES = Extracellular, extravascular volume fraction, fVASC = vascular volume fraction. fobj = objective function. fIC, fEES and fVASC are all fractions, which add to 1. Cellularity is number of cells per voxel, with units of cells/μm3. Objective function highlighting the ‘goodness of fit’ for the VERDICT model

The parametric maps generated from the VERDICT scans produce measurements of the intracellular volume fraction (fIC), cell radius (R), cellularity, extravascular extracellular volume fraction (fEES) and vascular volume fraction (fVasc). We also retain the fitting chi-squared objective function (fobj), which is a sum of square differences adjusted to account for offset Rician noise bias, as in [15, 16], to confirm successful fitting of the biophysical VERDICT model has been or highlight regions where the model is not appropriate. A typical example of such parameter maps is provided in Fig. 2.

#### Reporting of mp-MRI and VERDICT MRI

MRI examination reports should record the suspicion of cancer using an ordinal Likert scale (1 to 5): 1- tumour highly unlikely, 2- tumour unlikely, 3- equivocal, 4- tumour likely and 5- tumour highly likely. Strong evidence from multiple institutions confirms mp-MRI is able to accurately detect and localise ≥0.5 cc prostate cancer ≥ Gleason 4 [19–21].

The first 50 patients VERDICT MRI studies will be used to familiarise radiologists with VERDICT MRI derived parameter maps, as they ascend the learning curve. Radiologists will be allowed to review the VERDICT MRI with access to biopsy results for correlation once available. Potential conclusions drawn from VERDICT datasets will not be included in clinical MRI reports as at this stage we will not know the sensitivity or specificity of VERDICT. These patients will not form part of the main trial cohort.

A locked sequential read report for mp-MRI prior to and following evaluation of VERDICT MRI will be performed for the main trial cohort. mp-MRI results will be made available to the clinical team as per standard practice. VERDICT MRI results will be collected using a case report form but will not be revealed to the clinical care team so as not to negatively influence patient care. A radiologist will compare in vivo MR images and note areas of abnormality as defined by the conventional mp-MRI, and corresponding regions of interest (ROIs) on the parametric VERDICT maps. In the case of prostatectomy specimens, MR slices will be visually registered to the pathological specimen. For biopsies targeted using MRI, the lesion location can be ascertained from the operation note/pathology report and in the case of positive cores, specimens can be considered to be a successful target.

Quantitative measurements of vascular volume fraction, extracellular extravascular volume fraction, intracellular volume fraction, cell radius and cell density will be derived from VERDICT for correlation against histological measures (see section 3.4.3).

#### Fluidic markers from blood and urine

Whole blood, serum, plasma and urine will be collected from all patients in the study using existing standard operating procedures (SOPs) and assayed for diagnostic markers (PCA3, AGR2 (Anterior gradient protein 2 homolog), SPON2 (spondin 2), TMPRSS2 (Transmembrane protease serine 2), EN2(Homeobox protein engrailed-2), MSMB(Beta-microseminoprotein), GDF15(Growth differentiation factor 15), SIK2 (Serine/threonine-protein kinase) and CD10(cluster of differentiation 10)). Protein markers in all matrices will be assayed on a MesoScale discovery (MSD) platform and deoxyribonucleic acid (DNA) will be extracted from whole blood to investigate 22 prognostic single nucleotide polymorphisms (SNPs) associated with aggressive disease. RNA for the PCA3 and TMPRSS2 quantification from urine will be extracted according to an SOP already developed in our laboratory. qPCR for PCA3, TMPRSS2, 3 control genes (TBP (TATA binding protein), SDH (succinate dehydrogenase), RPLP2 (60S acidic ribosomal protein P)) and PSA will be used in triplicate to quantify gene expression. During the pilot phase we will continue to horizon scan for new markers and have included scope to add 2 further markers as evidence comes to light and assays are developed e.g. GOLM1 (golgi membrane protein 1), NAALADL2 (N-Acetylated Alpha-Linked Acidic Dipeptidase-Like 2).

We will also extract exosomes from the serum and plasma (when possible) of patients to derive molecular tumour characteristics using fluorescence-lifetime imaging microscopy (FLIM) based measurements as well as analysis of exosomal micro RNA (miRNA) that are known to be associated with cell-to-cell communication and the development of cancer as well as immunosuppression leading to the development of further pre-metastatic niche. Functional blood-derived miRNAs have been recognised as potential robust biomarkers in the detection of various types of cancer. The ability to screen for these miRNAs and to perform FLIM of the epidermal growth factor receptor (ErbB) family members will add important prognostic and predictive information for diagnosis and stratification of patients to treatment. Finally, we will separate peripheral blood mononuclear cells (PBMCs) from whole blood of newly diagnosed prostate cancer patients to perform immunophenotyping of immune cell populations with an ultimate goal to provide multi-modality patient stratification.

### Defining reference standards

#### Biomarker panel: mp-MRI result

Since it is envisaged that diagnostic biomarker thresholds in the blood or urine will be able to predict a negative mp-MRI result, and act as a gatekeeper to effectively rationalise its use, conventional mp-MRI result will form the reference standard. Any lesion (Likert score 3 and above) will be considered to be a positive result. VERDICT MRI will not be considered as part of the reference standard for fluidic markers as the utility of VERDICT MRI remains unknown.

### VERDICT MRI: histology/mpMRI based reference standard

**Fig. 3:**
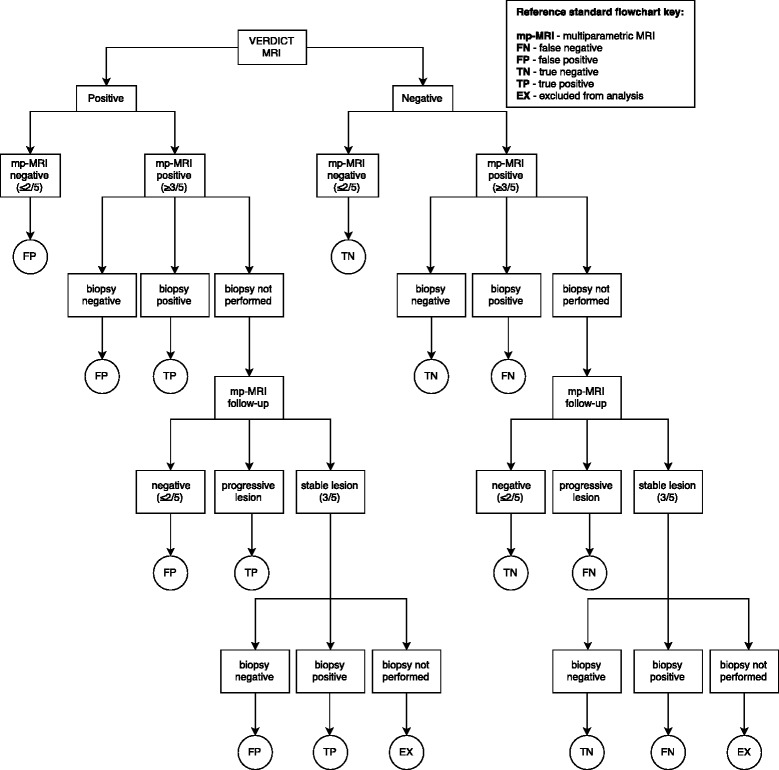
Derivation of reference standard flow chart

A lesion based reference standard will be derived (Fig. 3). mp-MRI has a 90-95 % negative predictive value for exclusion of aggressive disease [22] and will therefore form the reference for the index tests when mp-MRI is negative (Likert score 1-2/5). The positive predictive value of mp-MRI is limited and reported between 60-70 %. Therefore, where mp-MRI is positive (Likert score 3-5/5) a prostatectomy or biopsy will be performed if clinically appropriate. The prostatectomy or biopsy will then supersede the mp-MRI as the reference standard. Where a biopsy or prostatectomy is not performed, patients will be followed up with interval (6 months-1 year) mp-MRI as part of standard clinical care. A progressive Likert score (3/5 - > 4/5 or 5/5) or a progressive lesion (previously scored 4-5) on repeat mp-MRI will be considered as positive for the reference standard. A negative Likert score (1-2/5) on the repeat mp-MRI will be considered as negative for the reference standard. Lesions that remain stable with Likert score 3/5 will be deemed indeterminate and excluded from analysis unless biopsied. Based on previous internal audit, the total number of excluded patients is predicted to be approximately 10 %.

### Histopathological data processing and collection

The clinically most appropriate biopsy route for each patient will be used to obtain tissue, as informed by the mp-MRI and discussed and documented at the prostate Multi-disciplinary Team (MDT). Decision to biopsy or perform prostatectomy will be based on mp-MRI (not VERDICT MRI).

Tissue samples will be collected, fixed in formalin and embedded in paraffin. Sections will be and stained with hematoxylin and eosin (H&E) as per standard national health service (NHS) protocols. Immunohistochemical staining will also be performed for blood vessels and capillaries as per standard methods.

Histopathological assessment will be performed by two blinded histopathologists independently and then in consensus. Biopsy and whole block sections taken will be analysed after conventional H&E staining to assess tumor morphology including Gleason score, tumor volume/cancer core length, cell density, cell size distribution and percentage of epithelium/stroma. In addition immunohistochemistry for vascular markers will be performed for assessment of microvessel density.

To quantify the prostatic tissue components, automated segmentation of the core biopsies shall be performed, mapping blood vessels, lumen, epithelial cells and stroma using software developed in house.

In addition, detailed histological correlation will be sought for each of the specific imaging findings. A database table will be constructed listing the imaging observations and the histological findings listed in Table 4, with histological scores provided for each main observation.

**Table 4:** Imaging parameters vs. histological correlates

VERDICT parameter	Histological parameter
Intracellular volume fraction	Cell coverage fraction per high power field
Vascular volume fraction	Vascular coverage fraction per high power field
Extravascular extracellular volume fraction	Glandular + stromal coverage fraction per high power field
Cell radius	Average cell radius in a high power field
Cellularity	Cell count per high power field

### Statistical considerations

#### Sample size calculation

A sample size of 280 subjects achieves 80 % power to detect a difference of 0.1 between two diagnostic tests whose specificities are 0.7 and 0.6. This calculation uses a two-sided McNemar test with a significance level of 0.05. The prevalence of patients with no cancer or insignificant cancer (≤Gleason 3 + 3) is estimated at 0.6. The proportion of discordant pairs is estimated at 0.2. Allowing for 10 % of patients being excluded from the reference standard, a total of 365 patients (50 to allow radiologist training, followed by 315 patients for the main study) will be recruited. Based on current practice at our institution, approximately 10 mp-MRI studies are performed per week in men that meet the eligibility criteria. With a 50 % recruitment rate (note our audit data from previous similar studies supports a recruitment rate of 90 %), complete recruitment is expected to take 73 weeks.

### Outcome measures

All primary and secondary outcomes are presented in Table 5 below.

**Table 5:** Primary and secondary outcome measures

Primary outcome
Radiological assessment with added VERDICT MRI improves the diagnostic accuracy of mp-MRI for detection of significant prostate cancer by a minimum of 10 %
Secondary outcomes
• A group of diagnostic fluidic markers measured on the MesoScale discovery (MSD) platform and/or in DNA and RNA, can predict patients with a negative mp-MRI result (i.e. 1-2/5 Likert score).
• The use of patient serum-derived exosomes as ‘liquid biopsies’ for the identification of genomic and molecular aberrations that can be used to better predict patients with aggressive or high volume prostate cancer
• Technical validation of VERDICT:
○ VERDICT MRI is qualitatively and quantitatively repeatable
• Biological validation of VERDICT:
○ VERDICT cellularity measure correlates with histological cell density
○ VERDICT intracellular volume fraction correlates with segmented fractional histological intracellular component
○ VERDICT vascular volume fraction correlates with segmented fractional histological vascular component
○ VERDICT extracellular extravascular volume fraction correlates with fractional segmented histological glandular component + stromal component
• Set-up of imaging/fluidic marker outcome linked database

### Data analysis and outcome assessment

#### Fluidic markers

The diagnostic accuracy of fluidic markers will also be evaluated against the Likert score from the mpMRI, to gauge whether they may be used as a sensitive gatekeeper to reliably exclude patients in whom the mpMRI result is likely to be negative (Likert 1/2). To do this, results of each fluidic marker will be compared against the Likert score and a sensitivity and specificity will be acquired allowing for Receiver operating curve (ROC) and area under curve (AUC) analysis to subsequently be performed. Cancer volume and Gleason grade will be correlated with exosome levels, to judge whether they may have any useful clinical application as biomarkers in the future.

### VERDICT MRI

Lesion based analysis will be performed to compare specificity of mp-MRI with and without VERDICT MRI (at a Likert threshold of 3/5 as positive) against the reference standard, to ascertain whether VERDICT has any added diagnostic value. Correlation of VERDICT derived maps and quantitative histological parameters will also be assessed using correlation coefficients, and Bland-Altman plots.

Finally, a full clinical demographic, fluidic marker, qualitative and quantitative mp-MRI, and quantitative VERDICT parameter database will be established for future exploratory assessment and prediction of longer-term patient outcome.

We believe the INNOVATE study will be important, because it is one of the first clinical trials to bring together two important communities involved in prostate cancer research in a single project, namely imaging and fluidic biomarkers, who have traditionally worked in parallel. The findings of this study will also be particularly interesting, as the results from clinical trials of potential biomarkers are urgently needed and it also represents the world’s first clinical trial involving VERDICT MRI.

### Discussion

The INNOVATE study has some potential limitations. Firstly, as an observational trial, we are unable to take additional biopsies based on the VERDICT MRI result. This is because it would be unethical to perform additional biopsies at this stage of biomarker development, as it would lead to unnecessary increased risk.

However, if VERDICT MRI is shown to be successful in characterizing lesions within the prostate, additional biopsies would be particularly desirable where lesions are VERDICT positive but negative on conventional mpMRI, to determine whether such discrepancies are due to tumour.

Similarly, is also uncertain how many mp-MRIs will have lesions that are subsequently biopsied, as diagnostic and treatment decisions are made according to the standard clinical pathway. In addition, since PSA is a poor gatekeeper for MRI positive lesions, there will be a considerable number of scans which are mp-MRI negative, which could be said to increase the cost and reduce the efficiency of this trial, but will also allow us to better understand the appearances of normal VERDICT signal.

As with any quantitative imaging study testing a new sequence, the generalizability of data will be limited in the first instance, and will only apply to our scanner. However, if VERDICT is confirmed to be a repeatable and clinically useful test for the diagnosis and characterization of prostate cancer, our next step would be to conduct a reproducibility study, using the VERDICT scan protocol established on a different scanner. If the VERDICT sequence is confirmed to be acceptably reproducible, it would need to be programmed and made available on other scanners to confirm its usefulness as part of a multicenter trial. In this way, the development of the VERDICT sequence as a useful imaging biomarker should follow a logical stepwise progression, according to biomarker roadmaps, such as those outlined in the consensus document for use of diffusion-weighted MRI as a cancer biomarker [23], or by Cancer Research UK [24].

This study is also limited to using a combined histological/imaging/follow-up reference standard. Such standards are commonly employed in radiological studies when developing new techniques. Whilst tissue is usually preferable, it would be unethical to sample patients with no evident tumour at this stage of VERDICT development. Where tissue is obtained, there is some debate as to what forms the ideal histological reference standard. Whilst whole mount prostatectomy provides the most complete information with excellent spatial localization of tumors, which can later be registered to MRI datasets, prostatectomy cannot be used in all patients and therefore suffers from spectrum bias, whereby more aggressive tumors are selected [22]. Whilst template biopsy does not experience this problem, registration of the biopsy co-ordinates with the MRI is limited, and as a sampling technique is subject to sampling error [25], and may miss smaller tumors <0.2 cc [26]. Despite these controversies, both prostatectomy and template biopsy remain preferable to TRUS biopsy, which remains the standard of care in most centers but systematically misses 20 – 30 % of clinically significant cancers [27], particularly in the anterior gland [28].

## Conclusion

INNOVATE is a 365 patient cohort study being carried out over 3 years, whereby we wish to validate a biomarker panel to act as an effective gatekeeper to rationalize mp-MRI for widespread NHS adoption. We aim to confirm for the first time that VERDICT MRI is a repeatable technique and consider whether it can provide additional sensitivity and specificity for the detection of prostate cancer. If the parametric maps generated from VERDICT are shown to correlate with Gleason grade better than current quantitative multiparametric MRI measurands, VERDICT MRI could prove useful in a range of circumstances including the prevention or triggering prostate biopsy in biopsy naïve patients, patients being monitored under active surveillance and when assessing for disease recurrence following surgical, focal or radiotherapy.

### Trial status

Investigators from UCLH designed the trial and UCLH acts as the study sponsor. The UCLH Joint Research Office maintains responsibility for monitoring of Good Clinical Practice within the trial. A trial management group for the study comprises specialists from the disciplines of Radiology, Radiography and Biomarker science. Currently INNOVATE is open for recruitment in 1 Centre in the United Kingdom. Recruitment commenced in April 2016 and is expected to finish in March 2019. INNOVATE received UK Research Ethics Committee approval on 23rd December 2015 by the NRES Committee London—Surrey Borders with REC reference 15/LO/0692.﻿ INNOVATE is published on clinicaltrials.gov [29].﻿

## Additional file

Additional file 1: A referenced list of the fluidic biomarkers to be tested in the cohort. (DOCX 163 kb)

## Abbreviations

ADC: Apparent diffusion coefficient
AGR 2: Anterior gradient protein 2 homolog
AUC: Area under curve
BPH: Benign prostatic hyperplasia
CD10: Cluster of differentiation 10
DCE: Dynamic contrast enhanced imaging
DNA: Deoxyribonucleic acid
DRE: Digital rectal examination
DWI: Diffusion-weighted imaging
EN2: Homeobox protein engrailed-2
ErbB: Epidermal growth factor receptor
fEES: Extravascular extracellular volume fraction
fIC: Intracellular volume fraction
FLIM: Fluorescence-lifetime imaging microscopy
fobj: Objective function
fVasc: Vascular volume fraction
GDF15: Growth differentiation factor 15
GOLM 1: Golgi membrane protein 1
H&E: Hematoxylin and eosin
MDT: Multi-disciplinary team
miRNA: micro RNA
mp-MRI: Multi-parametric magnetic resonance imaging
MRD: MesoScale discovery
MRI: Magnetic resonance imaging
MSMB: Beta-microseminoprotein
NAALADL2: N-acetylated alpha-linked acidic dipeptidase-like 2
NAV: Number of averages
NHS: National Health Service
NICE: National Institute of Clinical Excellence
PBMC: Peripheral blood mononuclear cell
PCA3: Prostate cancer antigen 3
PSA: Prostate specific antigen
R: Cell radius
RNA: Ribonucleic acid
ROC: Receiver operating curve
ROI: Region of interest
RPLP2: 60S acidic ribosomal protein P
SDH: Succinate dehydrogenase
SIK2: Serine/threonine-protein kinase
SNP: Single nucleotide polymorphism
SOP: Standard operating procedure
SPON2: Spondin 2
T2W: T2 weighted imaging
TBP: TATA binding protein
TE: Echo time
TMPRSS2: Transmembrane protease serine 2
TRUS: Transrectal ultrasound
VERDICT: Vascular and extracellular restricted diffusion for cytometry in tumours
