# Supplementary material for: INNOVATE: A prospective cohort study combining serum and urinary biomarkers with novel diffusion-weighted magnetic resonance imaging for the prediction and characterization of prostate cancer
Source: BMC Cancer. 2016 Oct 21;16:816. doi: 10.1186/s12885-016-2856-2 (PMC5073433; doi:10.1186/s12885-016-2856-2)
Supplement: Additional file 1: — A referenced list of the fluidic biomarkers to be tested in the cohort. (DOCX 163 kb) [file 12885_2016_2856_MOESM1_ESM.docx]

Additional file 1

| **Biomarker name** | **Type** | **Matrix** | **Proven use** | **Stage of assay development** | **Tested in combination with mp-MRI** | **References** |
| --- | --- | --- | --- | --- | --- | --- |
| MSMB | Protein and SNP | Whole blood, serum, plasma or urine | Diagnostic and prognostic | Fully validated | No | ([1-5](#_ENREF_1)) |
| PCA3 | Non-coding RNA | Urine | Diagnostic | Being validated | Yes - additive | ([6-11](#_ENREF_6)) |
| GDF-15 | Protein and SNP | Whole blood, serum, plasma or urine | Diagnostic and prognostic | Fully validated | No | ([12-17](#_ENREF_12)) |
| CD10 | Protein | Serum, plasma or urine | Diagnostic and prognostic | Fully validated | No | ([18-21](#_ENREF_18)) |
| AGR2 | Protein | Serum, plasma or urine | Diagnostic | In validation | No | ([22-28](#_ENREF_22)) |
| SPON2 | Protein | Serum, plasma or urine | Diagnostic | In development | No | ([29-32](#_ENREF_29)) |
| TMPRSS2 | RNA | Urine | Diagnostic | In development | No | ([33-35](#_ENREF_33)) |
| EN2 | Protein | Urine | Diagnostic | Development initiated | No | ([36-39](#_ENREF_36)) |
| Aggressiveness SNP panel | DNA | Whole blood | Prognostic | Validated by collaborator | No | ([40](#_ENREF_40), [41](#_ENREF_41)) |
| SIK2 | Protein | Plasma | Diagnostic | Validated by HW lab and collaborator | No | (42) |

Table 6: A list of fluidics tests to be applied to the cohort.

1. Whitaker HC, Kote-Jarai Z, Ross-Adams H, Warren AY, Burge J, George A, et al. The rs10993994 risk allele for prostate cancer results in clinically relevant changes in microseminoprotein-beta expression in tissue and urine. PLoS One. [Research Support, N.I.H., Extramural

Research Support, Non-U.S. Gov't]. 2010;5(10):e13363.

2. Dahlman A, Rexhepaj E, Brennan DJ, Gallagher WM, Gaber A, Lindgren A, et al. Evaluation of the prognostic significance of MSMB and CRISP3 in prostate cancer using automated image analysis. Mod Pathol. [Research Support, Non-U.S. Gov't]. 2011 May;24(5):708-19.

3. Mhatre DR, Mahale SD, Khatkhatay MI, Desai SS, Jagtap DD, Dhabalia JV, et al. Development of an ELISA for sPSP94 and utility of the sPSP94/sPSA ratio as a diagnostic indicator to differentiate between benign prostatic hyperplasia and prostate cancer. Clin Chim Acta. [Research Support, Non-U.S. Gov't]. 2014 Sep 25;436:256-62.

4. Ahn J, Kibel AS, Park JY, Rebbeck TR, Rennert H, Stanford JL, et al. Prostate cancer predisposition loci and risk of metastatic disease and prostate cancer recurrence. Clin Cancer Res. 2011 Mar 1;17(5):1075-81.

5. Shui IM, Lindstrom S, Kibel AS, Berndt SI, Campa D, Gerke T, et al. Prostate cancer (PCa) risk variants and risk of fatal PCa in the National Cancer Institute Breast and Prostate Cancer Cohort Consortium. Eur Urol. [Research Support, N.I.H., Extramural

Research Support, N.I.H., Intramural

Research Support, Non-U.S. Gov't]. 2014 Jun;65(6):1069-75.

6. de Kok JB, Verhaegh GW, Roelofs RW, Hessels D, Kiemeney LA, Aalders TW, et al. DD3(PCA3), a very sensitive and specific marker to detect prostate tumors. Cancer Res. 2002 May 1;62(9):2695-8.

7. Hessels D, Schalken JA. The use of PCA3 in the diagnosis of prostate cancer. Nat Rev Urol. [Review]. 2009 May;6(5):255-61.

8. Leyten GH, Wierenga EA, Sedelaar JP, van Oort IM, Futterer JJ, Barentsz JO, et al. Value of PCA3 to Predict Biopsy Outcome and Its Potential Role in Selecting Patients for Multiparametric MRI. Int J Mol

Sci. 2013;14(6):11347-55.

9.Panebianco V, Sciarra A, De Berardinis E, Busetto GM, Lisi D, Buonocore V, et al. PCA3 urinary test versus 1H-MRSI and DCEMR in the detection of prostate cancer foci in patients with biochemical alterations. Anticancer Res. [Comparative Study]. 2011 Apr;31(4):1399-405.

10. Ploussard G, Durand X, Xylinas E, Moutereau S, Radulescu C, Forgue A, et al. Prostate cancer antigen 3 score accurately predicts tumour volume and might help in selecting prostate cancer patients for active surveillance. Eur Urol. 2011 Mar;59(3):422-9.

11. Sciarra A, Panebianco V, Cattarino S, Busetto GM, De Berardinis E, Ciccariello M, et al. Multiparametric magnetic resonance imaging of the prostate can improve the predictive value of the urinary prostate cancer antigen 3 test in patients with elevated prostate-specific antigen levels and a previous negative biopsy. BJU Int. [Randomized Controlled Trial]. 2012 Dec;110(11):1661-5.

12. Brown DA, Stephan C, Ward RL, Law M, Hunter M, Bauskin AR, et al. Measurement of serum levels of macrophage inhibitory cytokine 1 combined with prostate-specific antigen improves prostate cancer diagnosis. Clin Cancer Res. [Research Support, Non-U.S. Gov't]. 2006 Jan 1;12(1):89-96.

13. Nakamura T, Scorilas A, Stephan C, Yousef GM, Kristiansen G, Jung K, et al. Quantitative analysis of macrophage inhibitory cytokine-1 (MIC-1) gene expression in human prostatic tissues. Br J Cancer. 2003 Apr 7;88(7):1101-4.

14. Rasiah KK, Kench JG, Gardiner-Garden M, Biankin AV, Golovsky D, Brenner PC, et al. Aberrant neuropeptide Y and macrophage inhibitory cytokine-1 expression are early events in prostate cancer development and are associated with poor prognosis. Cancer Epidemiol Biomarkers Prev. [Research Support, Non-U.S. Gov't]. 2006 Apr;15(4):711-6.

15. Selander KS, Brown DA, Sequeiros GB, Hunter M, Desmond R, Parpala T, et al. Serum macrophage inhibitory cytokine-1 concentrations correlate with the presence of prostate cancer bone metastases. Cancer Epidemiol Biomarkers Prev. [Research Support, N.I.H., Extramural

Research Support, Non-U.S. Gov't]. 2007 Mar;16(3):532-7.

16. Stark JR, Wiklund F, Gronberg H, Schumacher F, Sinnott JA, Stampfer MJ, et al. Toll-like receptor signaling pathway variants and prostate cancer mortality. Cancer Epidemiol Biomarkers Prev. [Research Support, N.I.H., Extramural]. 2009 Jun;18(6):1859-63.

17. Yip PY, Kench JG, Rasiah KK, Benito RP, Lee CS, Stricker PD, et al. Low AZGP1 expression predicts for recurrence in margin-positive, localized prostate cancer. The Prostate. [Research Support, Non-U.S. Gov't]. 2011 Nov;71(15):1638-45.

18. Carlsson L, Ronquist G, Eliasson R, Egberg N, Larsson A. Flow cytometric technique for

determination of prostasomal quantity, size and expression of CD10, CD13, CD26 and CD59 in human seminal plasma. Int J Androl. [Evaluation Studies

Research Support, Non-U.S. Gov't]. 2006 Apr;29(2):331-8.

19. Dall'Era MA, True LD, Siegel AF, Porter MP, Sherertz TM, Liu AY. Differential expression of CD10 in prostate cancer and its clinical implication. BMC Urol. [Comparative Study

Research Support, N.I.H., Extramural]. 2007;7:3.

20. Fleischmann A, Schlomm T, Huland H, Kollermann J, Simon P, Mirlacher M, et al. Distinct subcellular expression patterns of neutral endopeptidase (CD10) in prostate cancer predict diverging clinical courses in surgically treated patients. Clin Cancer Res. [Research Support, Non-U.S. Gov't]. 2008 Dec 1;14(23):7838-42.

21. Voutsadakis IA, Vlachostergios PJ, Daliani DD, Karasavvidou F, Kakkas G, Moutzouris G, et al. CD10 is inversely associated with nuclear factor-kappa B and predicts biochemical recurrence after radical prostatectomy. Urol Int. 2012;88(2):158-64.

22. Bu H, Bormann S, Schafer G, Horninger W, Massoner P, Neeb A, et al. The anterior gradient 2 (AGR2) gene is overexpressed in prostate cancer and may be useful as a urine sediment marker for prostate cancer detection. The Prostate. [Research Support, Non-U.S. Gov't]. 2011 May;71(6):575-87.

23. Ho ME, Quek SI, True LD, Morrissey C, Corey E, Vessella RL, et al. Prostate cancer cell phenotypes based on AGR2 and CD10 expression. Mod Pathol. [Research Support, N.I.H., Extramural]. 2013 Jun;26(6):849-59.

24. Maresh EL, Mah V, Alavi M, Horvath S, Bagryanova L, Liebeskind ES, et al. Differential

expression of anterior gradient gene AGR2 in prostate cancer. BMC Cancer. [Research Support, N.I.H., Extramural]. 2010;10:680.

25. Wang Z, Hao Y, Lowe AW. The adenocarcinoma-associated antigen, AGR2, promotes tumor growth, cell migration, and cellular transformation. Cancer Res. [Research Support, N.I.H., Extramural

Research Support, Non-U.S. Gov't]. 2008 Jan 15;68(2):492-7.

26. Wayner EA, Quek SI, Ahmad R, Ho ME, Loprieno MA, Zhou Y, et al. Development of an ELISA to detect the secreted prostate cancer biomarker AGR2 in voided urine. The Prostate. [Comparative Study

Research Support, N.I.H., Extramural]. 2012 Jun 15;72(9):1023-34.

27. Zhang JS, Gong A, Cheville JC, Smith DI, Young CY. AGR2, an androgen-inducible secretory protein overexpressed in prostate cancer. Genes Chromosomes Cancer. [Research Support, N.I.H., Extramural

Research Support, U.S. Gov't, P.H.S.]. 2005 Jul;43(3):249-59.

28. Zhang Y, Forootan SS, Liu D, Barraclough R, Foster CS, Rudland PS, et al. Increased expression of anterior gradient-2 is significantly associated with poor survival of prostate cancer patients. Prostate Cancer Prostatic Dis. [Research Support, Non-U.S. Gov't

Research Support, U.S. Gov't, Non-P.H.S.]. 2007;10(3):293-300.

29. Edwards S, Campbell C, Flohr P, Shipley J, Giddings I, Te-Poele R, et al. Expression analysis onto microarrays of randomly selected cDNA clones highlights HOXB13 as a marker of human prostate cancer. Br J Cancer. [Comparative Study

Research Support, Non-U.S. Gov't]. 2005 Jan 31;92(2):376-81.

30. Lucarelli G, Rutigliano M, Bettocchi C, Palazzo S, Vavallo A, Galleggiante V, et al. Spondin-2, a secreted extracellular matrix protein, is a novel diagnostic biomarker for prostate cancer. J Urol. [Comparative Study]. 2013 Dec;190(6):2271-7.

31. Qian X, Li C, Pang B, Xue M, Wang J, Zhou J. Spondin-2 (SPON2), a more prostate-cancer-specific diagnostic biomarker. PLoS One. [Research Support, Non-U.S. Gov't]. 2012;7(5):e37225.

32. Romanuik TL, Ueda T, Le N, Haile S, Yong TM, Thomson T, et al. Novel biomarkers for prostate cancer including noncoding transcripts. Am J Pathol. [Research Support, N.I.H., Extramural

Research Support, Non-U.S. Gov't]. 2009 Dec;175(6):2264-76.

33. Schalken J, Dijkstra S, Baskin-Bey E, van Oort I. Potential utility of cancer-specific biomarkers for assessing response to hormonal treatments in metastatic prostate cancer. Ther Adv Urol. [Review]. 2014 Dec;6(6):245-52.

34. Tallon L, Luangphakdy D, Ruffion A, Colombel M, Devonec M, Champetier D, et al. Comparative evaluation of urinary PCA3 and TMPRSS2: ERG scores and serum PHI in predicting prostate cancer aggressiveness. Int J Mol Sci. 2014;15(8):13299-316.

35. Trock BJ. Circulating biomarkers for discriminating indolent from aggressive disease in prostate cancer active surveillance. Curr Opin Urol. [Research Support, N.I.H., Extramural

Research Support, U.S. Gov't, Non-P.H.S.

Review]. 2014 May;24(3):293-302.

36. Killick E, Morgan R, Launchbury F, Bancroft E, Page E, Castro E, et al. Role of Engrailed-2 (EN2) as a prostate cancer detection biomarker in genetically high risk men. Sci Rep. [Research Support, Non-U.S. Gov't]. 2013;3:2059.

37. Marszall MP, Sroka W, Adamowski M, Slupski P, Jarzemski P, Siodmiak J, et al. Engrailed-2 protein as a potential urinary prostate cancer biomarker: a comparison study before and after digital rectal examination. Eur J Cancer Prev. 2015 Jan;24(1):51-6.

38. McGrath SE, Michael A, Morgan R, Pandha H. EN2: a novel prostate cancer biomarker. Biomark Med. [Review]. 2013 Dec;7(6):893-901.

39. Morgan R, Boxall A, Bhatt A, Bailey M, Hindley R, Langley S, et al. Engrailed-2 (EN2): a tumor specific urinary biomarker for the early diagnosis of prostate cancer. Clin Cancer Res. [Research Support, Non-U.S. Gov't]. 2011 Mar 1;17(5):1090-8.

40. Karyadi DM, Zhao S, He Q, McIntosh L, Wright JL, Ostrander EA, et al. Confirmation of genetic variants associated with lethal prostate cancer in a cohort of men from hereditary prostate cancer families. Int J Cancer. 2014 Oct 1.

41. Lin DW, FitzGerald LM, Fu R, Kwon EM, Zheng SL, Kolb S, et al. Genetic variants in the LEPR, CRY1, RNASEL, IL4, and ARVCF genes are prognostic markers of prostate cancer-specific mortality. Cancer Epidemiol Biomarkers Prev. [Research Support, N.I.H., Extramural

Research Support, Non-U.S. Gov't]. 2011 Sep;20(9):1928-36.

42. Bon H, Wadhwa K, Schreiner A, Osborne M, Carroll T, Ramos-Montoya H, et al. Salt-inducible kinase 2 regulates mitotic progression and transcription in prostate cancer. Mol Cancer Res. 2015 Apr;13(4):620-35.
